# Role of bariatric surgery in reducing periprosthetic joint infections in total knee arthroplasty. A systematic review and meta-analysis

**DOI:** 10.1186/s12891-024-07288-2

**Published:** 2024-04-01

**Authors:** D. De Mauro, G. Balato, E. Festa, A. Di Cristo, L. Marasco, G. Loffredo, P. Di Lauro, D. Di Gennaro, G. Maccauro, D. Rosa

**Affiliations:** 1 Department of Public Health, Orthopedic Unit, “Federico II” University, Via Sergio Pansini, 5, Naples, 80130 Italy; 2 Department of Orthopedics and Geriatric Sciences, Catholic University of the Sacred Heart, Rome, Italy; 3 Department of Orthopedics and Rheumatological Sciences, Fondazione Policlinico Universitario A. Gemelli IRCCS, Rome, Italy

**Keywords:** Obesity, Bariatric surgery, Total knee arthroplasty, Periprosthetic joint infection

## Abstract

**Background:**

Obesity represents an epidemic of rising numbers worldwide year after year. In the Orthopedic field, obesity is one of the major causes leading to osteoarthritis needing Total Joint Arthroplasty (TJA). Still, contextually, it represents one of the most significant risk factors for joint replacement complications and failures. So, bariatric Surgery (BS) is becoming a valuable option for weight control and mitigating obesity-related risk factors. This review of the literature and meta-analysis aims to evaluate periprosthetic joint infections (PJI) and surgical site infections (SSI) rates in patients who underwent TKA after BS compared to obese patients without BS.

**Methods:**

Systematic review was performed according to Preferred Reporting Items for Systematic Review and Meta-Analyses (PRISMA) guidelines up to October 2023. We included longitudinal studies comparing obese patients who underwent total knee arthroplasty after bariatric surgery (study group) and obese patients who underwent TKA (control group). The surgical site infection and Periprosthetic joint infection rate were compared among groups using a meta-analytical approach.

**Results:**

The online database and references investigation identified one hundred and twenty-five studies. PJI rate differed significantly among groups, (z = -21.8928, *p* < 0.0001), with a lower risk in the BS group (z = -10.3114, *p* < 0.0001), for SSI, instead, not statistically significance were recorded (z = -0.6784, *p* = 0.4975).

**Conclusions:**

The current Literature suggests that Bariatric Surgery can reduce infectious complications in TKA, leading to better outcomes and less related costs treating of knee osteoarthritis in obese patients.

## Background

Obesity represents an epidemic plague, rising its numbers worldwide year after year [1]. High Body Mass Index (BMI) patients have increased risk of developing several diseases, such as diabetes, hypertension, and joint arthritis [2]. Obesity stands as a paradoxical factor in Orthopedics due to its role as one of the major causes leading to a Total Joint Arthroplasty (TJA), and contextually, one of the most significant risk factors for joint replacement complications and failures [3, 4]. International guidelines suggest joint replacement for patients with BMI > 40 kg/m2 should be avoided because the risk of postoperative complications may outweigh the benefit of the TJA [5]. Thus, surgeons must aim to induce BMI reduction in patients before the procedure [2]. Among tools for weight control, Bariatric Surgery (BS) is becoming a valuable option for those patients who present morbid obesity. Literature is still unclear about its use in Orthopedic patients, with conflicting results regarding complications rates and re-revisions incidence [6]. Although mechanical failures and post-operative re-admissions were often analyzed, BS’s impact on Periprosthetic Joint Infection (PJI) rates remains unclear. PJI still represents the most feared complication in prosthetic surgery [7–10], and its higher incidence in obese patients was assessed and confirmed in several studies. The number of papers relating TKA complications and bariatric surgery before TKA is increasing [11–14]. Given the benefits of BS for patients’ general health, it is necessary to establish if weight loss provided by metabolic surgery can also lead to an overall decrease of infection rates, both in PJI and surgical site infection (SSI). This review of the literature and meta-analysis aims to evaluate infections and SSI rates in patients who underwent TKA after BS compared to obese patients without BS.

## Materials and methods

### Search strategy and eligibility criteria

Systematic review was performed according to Preferred Reporting Items for Systematic Review and Meta-Analyses (PRISMA) guidelines [15] up to October 2023. Research papers investigating clinical outcomes and complications rate in patients treated with bariatric surgery before or after total knee arthroplasty were conducted in three different online databases: MEDLINE, Web of Science, Scopus. The keywords used for the research were combined as follow: (“BS” OR “Bariatric surgery” OR “metabolic surgery”) AND (“TKA” OR “TKR” OR “Total Knee Arthroplasty” OR “Total Knee Replacement”) AND (“Infection” OR “PJI” OR “SSI”).

All articles written in English were included, with no limitations for date of publication. Reference lists of selected articles were searched for additional articles that were not identified in the database search. Longitudinal studies (retrospective and prospective) and randomized controlled trials were evaluated and added to the final reference list. The exclusion criteria required the omission of case reports, expert opinions, prior systematic reviews, letters to the editor, studies that did not include patients who underwent TKA, studies that included different total joint arthroplasty (such as hip arthroplasty, shoulder etc.) and wherein TKA data could not be extrapolated. Studies not including infectious complications in the post-operative analysis were also excluded.

### Study assessment and data extraction

Initially, the titles and abstracts of the studies were screened by two independent reviewers. Full text was obtained for all abstracts that appeared to meet the inclusion criteria or those with any uncertainty. Then, each study was assessed based on the inclusion criteria by two independent reviewers, and the evaluation of the article by the senior author resolved any disagreement regarding the inclusion of any study. Relevant data were extracted from each study. Data describing participant demographics, sample size, and failure rate regarding SSI and PJI were recorded. The methodological quality of the studies included in this meta-analysis was assessed. The methodological quality of the studies included in this meta-analysis was assessed using the Methodological Index for Non-Randomized Studies (MINORS) score that yields a maximum score of 16 and 24, respectively, for non-comparative and comparative studies [16]. Two authors independently determined the MINORS score; the final score was obtained through consensus.

### Statistical analysis

The comparison between the infection rate in the study groups and the infection rate in the control group with a 95% confidence interval (CI) represented the primary outcome. Secondary outcomes were the rate of surgical site infections at follow-up in both groups. The analysis was carried out using the log odds ratio as the outcome measure. A random-effects model was fitted to the data. The amount of heterogeneity (i.e., τ²), was estimated using the restricted maximum-likelihood estimator [17]. In addition to the estimate of τ², the I² statistic are reported. In case any amount of heterogeneity is detected (i.e., τ² > 0), a prediction interval for the true outcomes is also provided. Studentized residuals and Cook’s distances are used to examine whether studies may be outliers and/or influential in the context of the model. Studies with a studentized residual larger than the 100 × (1–0.05/(2 X k))th percentile of a standard normal distribution are considered potential outliers (i.e., using a Bonferroni correction with two-sided alpha = 0.05 for k studies included in the meta-analysis). Studies with a Cook’s distance larger than the median plus six times the interquartile range of the Cook’s distances are influential. The rank correlation test and the regression test, using the standard error of the observed outcomes as predictors, are used to check for funnel plot asymmetry. Jamovi version 2.3 [18] and SPSS version 29 (SPSS, Chicago, IL, USA) for all statistical analyses. *P* ≤ 0.05 was considered significant.

## Results

**Fig. 1:**
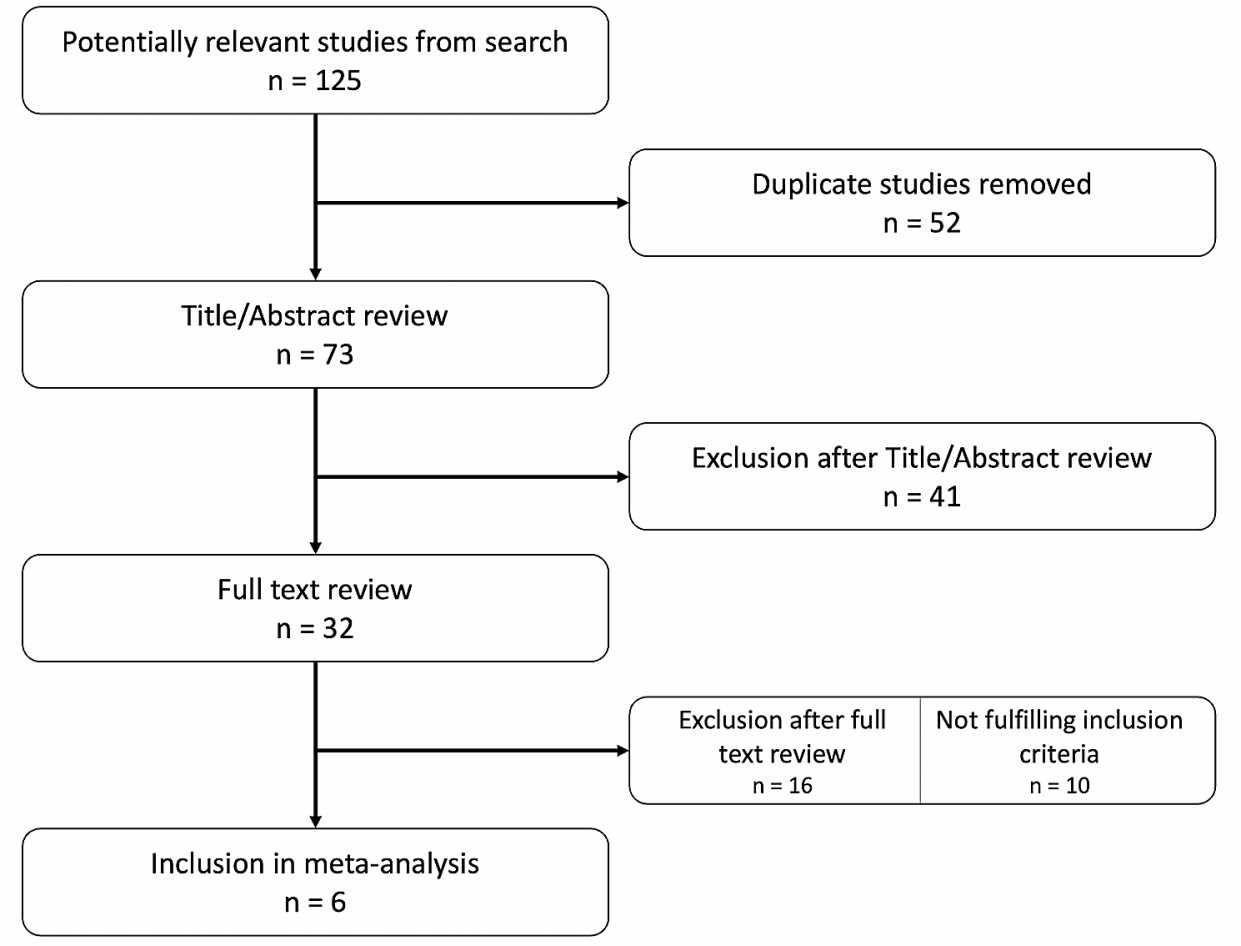
Study flowchart

A flow diagram of the search strategy is presented (Fig. 1). One hundred twenty-five studies were identified from the online database and reference investigation. After the duplicate removal and first screening based on titles and abstracts, the search produced a 32 papers list of full text to analyze. Six studies [19–24] were then included in the meta-analysis, 4 for the evaluation of PJI rate and 4 for the surgical site infection (SSI) rate (Table 1). Most of the studies were written and developed in USA, with only one other country represented (China).

A total amount of 3.045.096 patients was considered in the study, with a mean age of 60,1 years. The BS-group included 58.463 patients, 2.986.633 in the obese-group, instead. The mean percentage of patients with SSI in the study group and in the control, group was 4,2% and 3,9%, respectively, with a slightly lower rates of incidents in obese group. For what concerns the PJIs, the percentage in bariatric surgery group was lower (2.2%) than in control group (4.6%).

**Table 1:** Characteristics of the studies included in the systematic review and meta-analysis

Lead Author, Publication Date	Location	Journal	Mean Age	% Male	BS patients	% PJI	% SSI	Obese patients	% PJI	% SSI
Werner, 2014	U.S.A.	J. Arthroplasty	NS	26,8	219	1,8	1,4	11.294	5,0	1,8
McLawhorn, 2017	U.S.A.	J. Arthroplasty	57,0	17,6	2.636	1,5	NS	2.636	2,7	NS
Wang, 2019	China	J. Arthroplasty	NS	26,1	11.591	0,14	NS	87.335	0,2	NS
Meller, 2019	U.S.A.	JAAOS Glob Res Rev	NS	37,0	25.852	NS	1,0	2.675.575	NS	2,0
Sax, 2022	U.S.A.	J. Arthroplasty	61,5	27,0	17.960	5,3	5,5	209.383	10,6	5,3
Ryan, 2022	U.S.A.	J. Arthroplasty	62,0	18,0	205	NS	8,8	410	NS	6,4

NS: not stated

### Surgical site infections

**Fig. 2:**
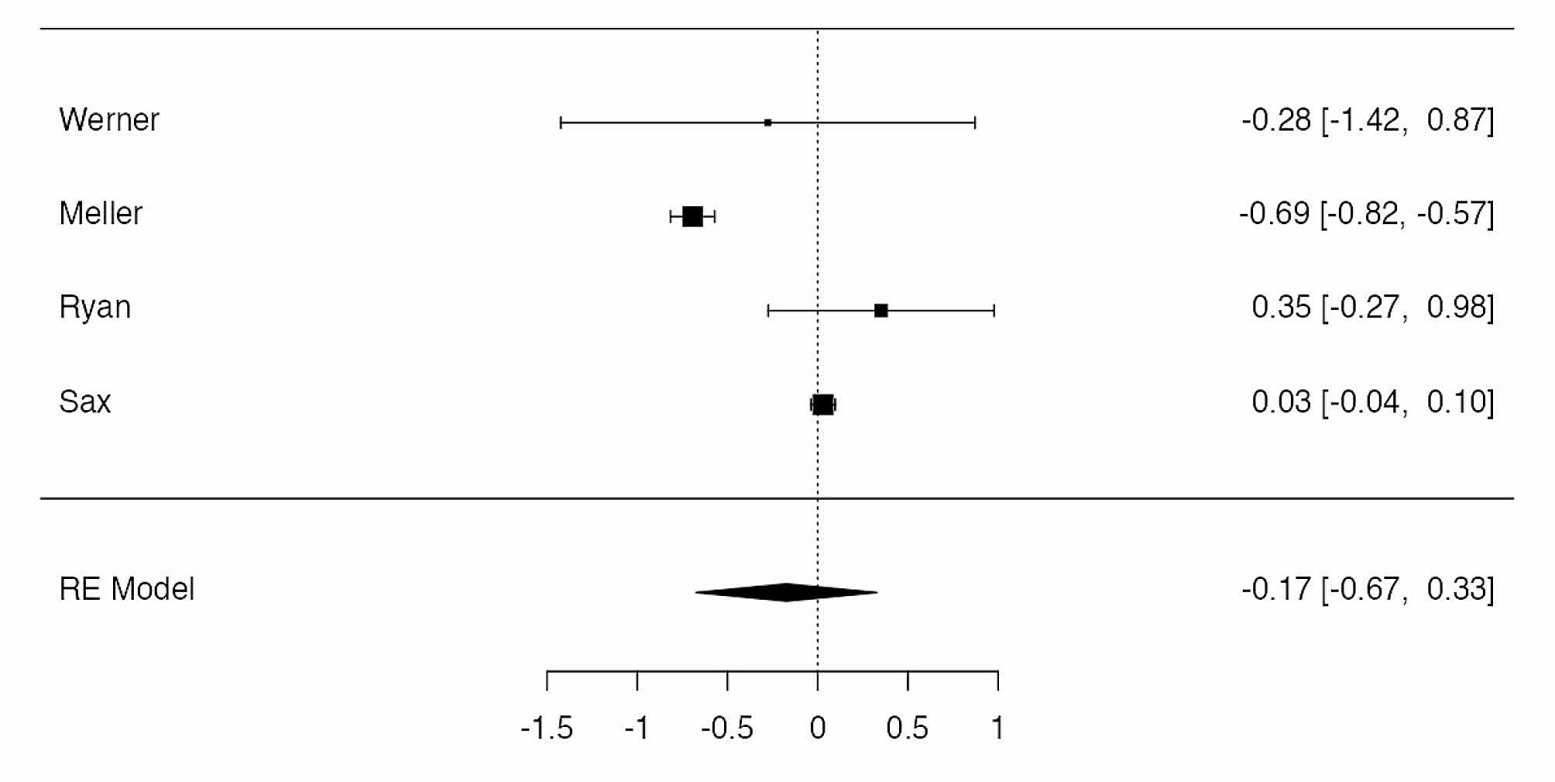
Rates of surgical site infections in patients treated by bariatric surgery before TKA and obese control group. The summary estimates presented were calculated using random-effects models; CI, confidence interval (bars)

Four studies were eligible for meta-analysis [19, 21, 22, 24], for a total amount of 44.236 patients in BS-group and 2.896.662 in obese group, with 1.265 and 64.879 cases of surgical site infection, respectively. The observed log odds ratios (OR) ranged from − 0.6936 to 0.3518, with the majority of estimates being negative. The estimated average log odds ratio based on the random-effects model was µ = -0.1734 (95% CI: -0.6742 to 0.3275). Therefore, the SSI rate did not differed significantly from zero (z = -0.6784, *p* = 0.4975). According to the Q-test, the true outcomes appear to be heterogeneous (I² = 96.44%, *p* < 0.0001). According to the Cook’s distances, none of the studies could be considered as overly influential. Neither the rank correlation nor the regression test indicated any funnel plot asymmetry (*p* = 0.7500 and *p* = 0.7011, respectively) (Fig. 2).

### Periprosthetic joint infections

**Fig. 3:**
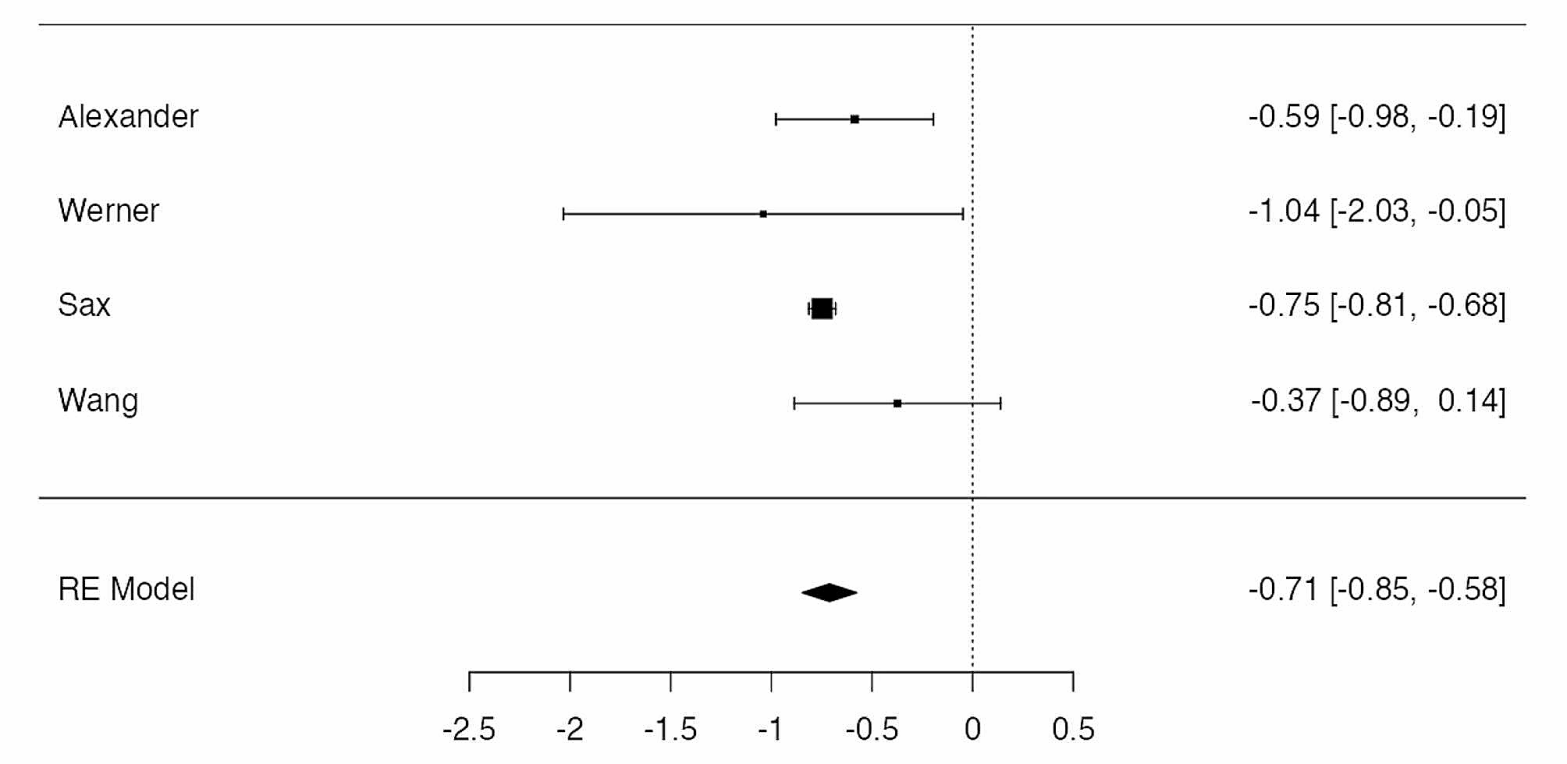
Rates of infection in patients treated by bariatric surgery before TKA and obese control group. The summary estimates presented were calculated using random-effects models; CI, confidence interval (bars)

Four studies were eligible for meta-analysis [19, 20, 22, 23], for a total amount of 32.406 patients in BS-group and 310.648 in obese group, with 1.003 and 23.027 cases of infections, respectively. The observed log odds ratios (OR) ranged from − 1.0405 to -0.3733, with the majority of estimates being negative. The estimated average log odds ratio based on the random-effects model was µ = -0.7110 (95% CI: -0.8462 to -0.5759). Therefore, the PJI rate differed significantly from zero (z = -10.3114, *p* < 0.0001), with a lower risk in the BS-group. According to the Q-test, there was no significant amount of heterogeneity in the true outcomes (I² = 11.69%, *p* = 0.398). A 95% prediction interval for the true outcomes is given by -0.9018 to -0.5203. Hence, even though there may be some heterogeneity, the true outcomes of the studies are generally in the same direction as the estimated average outcome. According to the Cook’s distances, none of the studies could be considered as overly influential. There was no indication of outliers in the context of this model. Neither the rank correlation nor the regression test indicated any funnel plot asymmetry (*p* = 0.750 and *p* = 0.387, respectively) (Fig. 3).

## Discussion

Patients with BMI > 40 Kg/m^2^ are unanimous considered as worst candidates to joint replacement, especially to total knee arthroplasty. Nevertheless, obese patients suffers more often than non-obese patients of knee osteoarthritis, leading to a surgical indication to knee replacement. Due to this paradoxical situation, in Literature are becoming more commons studies comparing TKA outcomes in obese patients treated with bariatric surgery and obese patients without BS [19–26]. Existing reviews of the literature about this topic [27, 28] consider mechanical failures and re-admissions into the Hospital for early complications as main endpoints, without focusing on infectious complications. For what we know, this is the first review of the literature and meta-analysis specifically about PJI and SSI rates in TKA. Therefore, the strength of this paper is solid and current about such a dramatic topic in daily activity of Orthopedic surgeon [8].

Smith et al. [28] collected cases from 5 studies, highlighting a significant difference in wound infection, with lower rates in BS patients. However, they performed a literature review and meta-analysis considering both TKA and total hip arthroplasty (THA), we opted, instead, to evaluate single TKA procedures, in order to compare homogeneous groups and same joint.

More recently, Yan et al. [27] provided a more updated review of the literature about this topic, involving more studies than us in the meta-analysis for infections (7). However, they considered all infectious complications under a general label “infection”, without going into details of the pathology. We analyzed two different outcomes, PJI and SSI, providing a meta-analysis for each of them.

The correct outcome to evaluate the risk of septic failures was chosen according to the data found in the Literature. First endpoint was to assess periprosthetic joint infection rates, considering it as the mean diagnosis when it comes to septic complications [29]. Surgical site infections, instead, were less related to actual septic failure, but the available data allowed us to use this information to better define the impact of bariatric surgery as prevention of infections in joint arthroplasty. The choice of performing a meta-analysis using random-effects models rather than fixed-effects, due to the heterogeneous data and the size of the studies, according to Nikolakopoulou et al. [30].

Our data allowed us to deliver a statistically significant statement about first outcome, not for the SSI rates, instead. Thus, obese patients underwent bariatric surgery before TKA seem to have a lower risk of PJI, compared to patients who underwent TKA without a BS before. SSI risk seems to be different, but *p*-value > 0.05.

This data suggests lack of correlation between Bariatric surgery and SSI risk in TKA, thus superficial wound infections cannot be addressed through BS, unlike the PJI.

As matter of facts, SSI includes partially those infections who later became deeper infections, often representing an early manifestation of PJI. Thus, even if the SSI-risk remain similar in both groups, it seems less probable for the infections to affect deeper layers and the prosthesis itself in BS-group, rather than in Obese-group, and this represents an important result of our meta-analysis.

Even if included studies are heterogeneous, none of them is overly influential compared to others, according to our analysis. Each paper contained enough data to reach inclusion criteria and make it to the final draft for the meta-analysis, considering surgical site infection and periprosthetic infection among complications in TKA.

Some studies [21, 24] offered a further comparison between BS-patients and non-obese patients, matching patients underwent bariatric surgery with patients with the same BMI they reached after the surgery. Due to the low numerosity of these studies, meta-analysis was not performed, but comparing the straight numbers, seems to be still higher infection risk in BS-group. This can suggest that BMI reduction alone is not able to grant an adequate return to pre-obesity condition. Further studies comparing obese, non-obese and BS patients are needed to better analyze relations among metabolic disorders, obesity and TKA complications.

Main limitation of the study is clearly the studies number, not allowing a more appropriate and accurate meta-analysis. All the included studies are, moreover, not specifically sets for infectious outcomes, but mostly on early complications and revisions. Thus, the data we analyzed were often not clear determined and highlighted. Bariatric Surgery can be a solid ally for orthopedic surgeons in facing joint osteoarthritis in obese patients, but more and stronger studies are needed, to allow this procedure to be included in treatment algorithm.

## Conclusions

The current Literature suggests that Bariatric Surgery can reduce infectious complications after TKA. Indeed, Obese patients treated with BS before TKA are less prone to surgical site infections and periprosthetic joint infections compared to morbidly obese patients.

## Data Availability

The datasets used and/or analysed during the current study are available from the corresponding author on reasonable request.

## Abbreviations

TJA: Total Joint Arthroplasty
TKA: Total Knee Arthroplasty
PJI: Periprosthetic Joint Infection
BS: Bariatric Surgery
SSI: Surgical site infection
CI: Confidence Interval
